# Application and clinical efficacy of Sufu medical chitosan hydrogel dressing in radiation skin damage caused by radiotherapy for cervical cancer

**DOI:** 10.12669/pjms.38.8.5569

**Published:** 2022

**Authors:** Xiaodan He, Xiufen Hu, Xiaojing Dong, Shenjie Li, Yali Ni

**Affiliations:** 1 Xiaodan He, Cancer Hospital of China Medical University, Liaoning Cancer Hospital & Institute, Shenyang 110042 Liaoning, China; 2 Xiufen Hu, Cancer Hospital of China Medical University, Liaoning Cancer Hospital & Institute, Shenyang 110042 Liaoning, China; 3 Xiaojing Dong, Cancer Hospital of China Medical University, Liaoning Cancer Hospital & Institute, Shenyang 110042 Liaoning, China; 4 Shenjie Li, Cancer Hospital of China Medical University, Liaoning Cancer Hospital & Institute, Shenyang 110042 Liaoning, China; 5 Yali Ni, Cancer Hospital of China Medical University, Liaoning Cancer Hospital & Institute, Shenyang 110042 Liaoning, China

**Keywords:** Cervical cancer, Sufu, Radiation dermatitis, Clinical efficacy

## Abstract

**Objectives:**

To investigate the benefits of Sufu medical chitosan hydrogel dressing(Sufu) in the prevention and control of radiation skin damage during radiotherapy for cervical cancer as a combined modality.

**Methods:**

Ninety-seven cervical cancer patients who underwent radiotherapy at the Cancer Hospital of China Medical University between May 2017 and November 2018 were recruited according to given inclusion and exclusion criteria. The patients were assigned to a control group (n=48, washing the perineal area with normal saline) and an observation group (n=49, application of Sufu onto the site of radiotherapy in addition to washing the perineal area with normal saline). The treatment regimens for the two groups continued until the end of radiotherapy. A comparison of the RTOG (Radiation Therapy Oncology Group) grading of acute radiation-induced skin reactions (ARISRs), pain intensity (measured by the verbal rating scale (VRS)) and post-treatment wound healing was drawn between the two groups.

**Results:**

In the observation group, 81.6% (40/49) of the patients had radiation dermatitis, which was significantly lower than the incidence rate (95.8%, 46/48) in the control group (P <0.05). The observation group was at higher risk of radiation dermatitis when given a high radiation dose, while the control group was more likely to have radiation dermatitis when administered with a moderate radiation dose (P <0.05). The median time of occurrence of pain and the median time of onset of skin reactions were significantly later in the observation group as compared with the control group (P <0.05, respectively). In the observation group, the pain relief rate was 92.50% at Day-3, and the wound healing rate was 95.0% at Day-7, significantly higher than in the control group (73.9% and 80.4%) (P <0.05, respectively).

**Conclusions:**

During radiotherapy for cervical cancer, Sufu can effectively prevent and control radiation-induced skin and mucous membrane damage, delay the onset of radiation dermatitis and substantially reduce the incidence rate, relieve radiation dermatitis and pain and promote wound healing.

## INTRODUCTION

Cervical cancer is the most important carcinoma that affects the female genital organs. 1,2 In principle, early treatment is followed by radiotherapy as the second phase of treatment. Therefore, radiotherapy is regarded as the most important part of cervical cancer treatment.^3^-^5^ However, radiotherapy for cervical cancer may lead to radiation repair and radiosynthesis. Besides, skin is usually hydrated and suppler on cloudy days and becomes more susceptible to radiation damage.^6^ Moreover, prevention and control of radiomaterial-induced skin damage should be timely implemented since there is a risk of infection due to turbulence that may affect the immune system; when an ulcer and infection occur, the treatment course will be adversely affected and even terminated, which causes patients extreme pain and suffering and means a greater financial burden on their shoulders.^7^,^8^ to avoid this, Sufu has recently gained widespread use in cervical cancer patients to prevent radiation skin damage.

## METHODS

Minety seven patients who were admitted by the Cancer Hospital of China Medical University and underwent radiotherapy for cervical cancer between May 2017 and November 2018 were included in this study.

### Ethical Approval:

The study was approved by the Institutional Ethics Committee of Cancer Hospital of China Medical University on October 20,2020 (No.:202035), and written informed consent was obtained from all participants and their family members.

### Inclusion criteria:

- Satisfied the diagnostic criteria for cervical cancer specified in the 2020 NCCN clinical practice guidelines (Version 1), was pathologically diagnosed with ≥ stage IIb cervical cancer and met the indications for radiotherapy;
- Had no medical history of skin diseases (e.g., rash) or cancer;
- Showed favorable tolerance to Sufu without any signs of allergy;
- Acted actively and cooperatively during nursing care.

### Exclusion criteria:

- Had a history of radiotherapy or related treatment for cancer;
- Had complications such as severe hepatic or renal dysfunction, cachectic organopathies, and organ failure;
- Had a skin defect in the perineal area, which was not cured completely upon recruitment;
- Was unable to cooperate during diagnosis and nursing care because of mental disorders or any other conditions;
- Refused to participate in this study. Patients (n =97) included in this study were assigned to an observation group (n =49) and a control group (n =48) using a random number table.

### Methods: Radiotherapy:

The two groups were administered with standard intensity-modulated radiation therapy (IMRT) as follows: CT positioning and a series of operations (including target volume delineation, radiation field design, and verification) were performed to develop an ideal radiotherapy program constituted by five weeks of wide-field radiotherapy (DT200 cGy/5 fractions) and intracavitary afterloading therapy (frequency & radiation dose: DT600-750 cGy/4-6 fractions) using a 192Ir afterloading system. In the control group, the perineal area was washed with normal saline twice daily (morning and evening) until the end of the radiotherapy program. Additional nursing care was provided for each patient to ensure the perineal area remained dry and clean and remind each patient of avoiding application of any irritant substances, wearing soft cotton underwear and not scratching the perineal area; in the meantime, a hygiene routine was established to maintain a dry, clean radiation field. In the observation group, an appropriate amount of Sufu was applied onto the radiation site in addition to the treatment with normal saline as provided for the control group, and the gel should be removed thoroughly before the next session of radiotherapy.

### Treatment for radiation skin damage:

Radiotherapy should be discontinued upon the occurrence of radiation dermatitis. In both groups, aseptic processing of the wound surface was completed by rinsing with normal saline, and direct application of Sufu was given 2-3 times per day; after the wound had healed, topical application of zinc ointment was prescribed. For patients with severe ulcers or plentiful secretions, their medical conditions should be evaluated, and if necessary, their dressings should be replaced more frequently to minimize the risk of bacterial infection; it should be noted that the dressings should be covered by gauze to ensure sterility of the wound.

### Evaluation criteria:

Comparison of radiation dermatitis grading: with the RTOG grading being the evaluation criteria, ARISRs were classified as several grades: Grade 0 (none): no change; Grade-I: faint or dull erythema, hair loss or dry, peeling skin; Grade-II: tender or bright erythema, patchy moist desquamation or moderately edematous skin; Grade-III: Confluent moist desquamation and pitting edema in areas except wrinkles; Grade-IV: ulceration, bleeding and necrosis. Comparison of radiation dose inducing radiation dermatitis; Comparison of time of occurrence of pain and time of onset of skin reactions; Comparison of pain relief and wound healing in patients with radiation dermatitis: the 0-to-3 verbal rating scale (VRS) was used for pain assessment, with Grade-II and above indicative of the presence of pain. Pain relief was assessed after three days of treatment: a downgrade by ≥2 or absence of pain was defined as a case of complete response (CR); a downgrade by ≥1 was regarded as a case of partial response (PR); no record of any improvement was classified as no response (NR). At Day-7, would healing was evaluated according to the following criteria: the wound was healing if the skin felt itchy, sagging, and erosion of skin tissue recovered; the wound was healing from around the surface of erosion if symptoms were relieved evidently or reduced without exudation; no visible remission of symptoms and presence of exudate or erosion were indicative of loss of wound strength.

### Statistical Methods:

The software SPSS20.0 (SPSS Inc., USA) was used for statistical analysis. Measurement data were represented by “mean ± standard deviation”, and those fitting normal distributions (i.e., time of occurrence of pain, time of onset of skin reactions, radiation dose) were examined by the t-test. Enumeration data (i.e., pain intensity, wound healing rate, staging) were examined by the chi-squared (χ2) test or the u-test. Significance was set at the level of P <0.05.

## RESULTS

The two groups showed no statistically significant differences in baseline characteristics (e.g., age, pathological type, FIGO staging) (P >0.05, respectively). Table-I.The incidence of radiation dermatitis was 81.6% (40/49) in the observation group, significantly lower than 95.8% (46/48) in the control group (P <0.05). Table-II.

**Table-I:** Comparison of general information

	Age	Pathological type			FIGO staging			Radiation dose (Gy)

		Squamous-cell carcinoma (SqCC)	Adenocarcinoma (AdC)	Adenosquamous carcinoma (ASC)	Others	Stage IIb	IIIb	
Observation group (n =49)	48.7±12.9	42	3	1	2	36	13	48.1±17.5
Control group (n =48)	49.8±14.2	44	2	1	1	34	14	43.8±16.2
t/χ2χ	0.400	0.580					0.084	0.566
P-value	0.690	0.901					0.772	0.573

**Table-II:** Comparison of radiation dermatitis grading

	RTOG grading of ARISRs					u-value	P-value

	Grade 0	Grade-I	Grade-II	Grade-III	Grade-IV		
Observation group (n =49)	9	26	10	3	1	4.525	0.000
Control group (n =48)	2	12	14	13	7		

In the observation group, a high radiation dose was associated with a greater risk of radiation dermatitis, while the control group became more susceptible to radiation dermatitis when given a moderate radiation dose (P <0.05). Table-III.The median time of occurrence of pain and the median time of onset of skin reactions were significantly later in the observation group as compared with the control group (P <0.05, respectively). Table-IV. At Day-3, the pain relief rate was 92.50% in the observation group, significantly higher than 73.9% in the control group; at Day-7, the wound healing rate was 95.0% in the observation group, significantly higher than 80.4% in the control group (P <0.05). Table-V.

**Table-III:** Comparison of radiation dose inducing radiation dermatitis

	Radiation dose (Gy)			u-value	P-value

	<20 Gy	20-40 Gy	>40 Gy		
Observation group (n =40)	2	14	24	2.849	0.004
Control group (n =46)	10	21	15		

**Table-IV:** Comparison of time of occurrence of pain and time of onset of skin reactions.

	Median time of occurrence of pain	Time of onset of skin reactions
Observation group (n =40)	29.2±7.1	27.4±7.7
Control group (n =46)	21.8±8.3	23.1±6.5
t-value	4.722	2.969
P-value	0.000	0.004

**Table-V:** Comparison of pain relief and wound healing

	Pain relief at Day 3			ORR (%)	Wound healing at Day 7			ORR (%)
	
	CR	PR	NR		CR	PR	NR	
Observation group (n =40)	27	10	3	37(92.5)	26	12	2	38(95.0)
Control group (n =46)	20	14	12	34(73.9)	18	19	9	37(80.4)
u/χ2χ		2.502		5.133		2.615		4.069
P-value		0.012		0.024		0.009		0.044

## DISCUSSION

This study showed that topical application of Sufu onto the skin of the radiation site and the area(s) with radiation dermatitis was safe and effective to reduce the incidence of radiation dermatitis or alleviate radiation dermatitis as an ideal treatment.

Radiation dermatitis is one of the most common complications that occur in cervical cancer patients during radiotherapy.^9^-^11^ Since the perineal area has delicate and sensitive skin, irritations caused by urine and feces, frictions with clothes and radiation may induce swelling and spasm of the capillary walls, reactive dilation and local congestion of the capillaries, narrowing or obstruction of lumens and direct radiation damage, resulting in a markedly higher incidence of radiation dermatitis during radiotherapy for cervical cancer as compared with other types of cancer.^12^,^13^ Moreover, considering the presence of urine and feces and the humid environment in the perineal area, radiation dermatitis produces an enormous impact on cervical cancer patients subject to radiotherapy as it is highly likely to aggravate persistently and even gives rise to serious secondary infections that adversely affect the radiotherapy program and threaten patients’ health.^14^,^15^ Therefore, it is clinically significant to effectively prevent radiotherapy-related toxic and side effects (e.g., radiation dermatitis) and optimize treatments for such adverse events in cervical cancer cases.

Currently, topical application of medicine onto the radiation site is widely accepted as an effective treatment to prevent radiation dermatitis.^16^,^17^ Medicine on the surface of the radiation site provides protection for the wound by preventing infection and promoting epithelial growth.^18^ However, very few researchers have discussed the application of Sufu in combination with radiotherapy for cervical cancer and its effects and clinical value regarding prevention and control of radiation skin damage. Evidence shows that erythema, erosion of mucous membrane, and even ulceration may occur in the radiation or exposure field.^19^ Sufu is mainly formulated with chitosan and collagen and contains recombinant human epidermal growth factor (rhEGF). The key ingredients work synergistically to relieve pain, combat infection, promote wound healing and tissue recovery and retain moisture, thereby achieving effective prevention and control of radiation dermatitis.^20^,^21^ Sufu appears to follow these mechanisms: 1) offering local anti-inflammatory, antiviral, anti-radiation and antioxidant protection; 2) promoting wound healing by long-time retention and sustained release of nutritional ingredients (e.g., collagen) and growth factors that provide consistent stimulation for the cells in the skin or wound after topical application.^22^; 3) stimulating cytokines to reduce swelling and spasm of capillary walls, improve local blood circulation, support local oxygenation and blood supply and accelerate remission of local inflammatory edema; 4) forming a thin layer on the surface of the wound to maintain the humid environment of the wound and isolate the wound from bacteria, thereby minimizing the risk of infection and creating a favorable environment for granulation tissue to grow healthily.^23^

### Limitations:

It includes a small number of patients, short follow-up time. Moreover, nutritional indicators of patients before and after treatment have not been included in this study, single-center study, and no further analysis of cervical cancer patients with the same family social and economic factors. In response to this, proactive countermeasures will be taken to further increase the sample size, follow-up time, and analysis of the difference in the long-term incidence of cervical cancer under two treatment regimens in future studies

## CONCLUSION

During radiotherapy for cervical cancer, Sufu can effectively prevent and control radiotherapy-induced skin and mucosal damage, delay the onset of radiation dermatitis and substantially reduce its incidence, alleviate symptoms of radiation dermatitis, ease the pain and promote wound healing.

### Authors’ Contributions:

**XH &** **XH:** Designed this study, prepared this manuscript, are responsible and accountable for the accuracy and integrity of the work.

**XD &** **SL:** Collected and analyzed clinical data.

**YN:** Data analysis, Significantly revised this manuscript.
